# Supplementary material for: Comparative primary paediatric nasal epithelial cell culture differentiation and RSV-induced cytopathogenesis following culture in two commercial media
Source: PLoS One. 2020 Mar 26;15(3):e0228229. doi: 10.1371/journal.pone.0228229 (PMC7098550; doi:10.1371/journal.pone.0228229)
Supplement: Attachment — Submitted filename: Response to reviewers.docx [file pone.0228229.s001.docx]

Dear Prof. Tripp,

We would like to thank you and your reviewers of PLOS One for your timely and constructive responses to our submitted manuscript:

**“Comparative primary paediatric nasal epithelial cell culture differentiation and RSV-induced cytopathogenesis following culture in two commercial media”**

As requested, the anonymised data has been uploaded to an accessible database. The DOI is: [10.17034/7c15ce87-a011-47e7-ba55-50e0ccb550a9](https://doi.org/10.17034/7c15ce87-a011-47e7-ba55-50e0ccb550a9)

We thank the reviewers for noting the importance of this work, despite the simplicity of the study. Fundamental research into reagents and cell culture such as this enable decisions during future studies. We have addressed the editor and reviewer’s comments below and hope that the revised manuscript is now acceptable for publication.

Yours sincerely,

Lindsay Broadbent

**Response to editor’s and reviewer’s comments:**

Editor: *The study examines a small number subjects (n=3) and the experiment does not appear to be replicated as noted by a reviewer. The issues noted by the reviewers must be addressed, particularly the consistency of results among plates and among different days (replicates).*

**Response:** We recognise that the main point of concern for the editor and reviewers is the number of donors used and the replicates. We understand that n=3 is limited and ideally, we would increase this. However, it should be noted that these primary nasal epithelial cells are from neonatal donors within 10 days of life and, as such, are incredibly precious samples with limited number of cells. The use of these cultures, and indeed every Transwell, had to be meticulously reasoned. As the reviewer pointed out we have stated that this is a limitation of the study in the discussion.

We would also like to draw the editor’s attention to the reproducibility of the results within the different culture conditions, despite the expected donor-to-donor variation. In addition, the data from Transwells seeded at the two different seeding densities (2x10^4^ and 5x10^4^) were remarkably similar for cell counts and virus growth kinetics at the time of exploitation for these experiments (Figure 1B and Figure 4 of the manuscript) (Figure 1 below). Included below is the uninfected Transwell immunofluorescence data to highlight the similarities between the two seeding densities.

We have also included the ELISA data for IL-29 secretion following mock or RSV BT2a infection for Transwells derived from both seeding densities to show the remarkable similarity between these results (Figure 2 below). We are very confident, therefore, that these data are reproducible within our experimental conditions. We chose to present only the data derived from the seeding density of 5x10^4^ for some of the figures for simplicity and consistency of experimental conditions. We have mentioned this is the revised manuscript (Line 189). However, if the Editor and/or Reviewers require us to include the extra data from Transwell cultures seeded at 2x10^5^ cells, we would be happy to modify to manuscript accordingly.

Figure 1 for reviewers: WD-PNEC cultures (n=3 donors) with an initial seeding density of 2x10^4^ or 5x10^4^ per Transwell were differentiated in Promocell or PneumaCult medium. After 21 days cultures were fixed in 4% paraformaldehyde and stained for β-tubulin, a ciliated cell marker; Muc5ac, a goblet cell marker and counterstained for DAPI. The average number of total, ciliated and goblet cells from 5 fields of view per donor was calculated (left panel). The percentage of ciliated cells and goblet cells in the culture was calculated (right panel). Images were acquired using a Nikon Eclipse 90i at x60 magnification.

Figure 2 for reviewers: WD-PNEC cultures seeded at of 2x10^4^ (red) or 5x10^4^ (black) per Transwell (n=3 donors) were differentiated in Promocell or Pneumacult medium. After 21 days cultures were infected with RSV BT2a or mock infected. Basolateral medium was harvested and snap frozen every 24 hpi. The concentration of IFNλ1/IL-29 in the basolateral medium at 96 hpi was quantified by ELISA

Reviewer 1 states: ‘*This is particularly true of the conclusion drawn that a greater degree of (pseudo?) stratification of the cultures differentiated was achieved in the PneumaCult medium compared to Promocell (data not shown).’*

**Response:** We have modified the language (now Line 171) to avoid over emphasis on the differences in orthogonal sections to read: ‘*Orthogonal sections suggest a greater degree of stratification of the cultures differentiated in PneumaCult medium compared to Promocell medium.* Understanding the apparent greater stratification would require much more extensive work and would constitute a new project/manuscript.

Reviewer 1 states: ‘*unknown proprietary constituents in Pneumacult media, which is not trivial (lines 273-283).’*

**Response:** We thank the reviewer for emphasising this point and have stated this is the manuscript.

Reviewer 2 states: ‘*The important finding is that both culture systems generated many non-significant effects as pointed out including RSV titers. The weakness is that the manuscript emphasizes the few differences.’*

**Response:** We have modified the language in the discussion to indicate that there are several similarities between cultures grown using the different media. The paragraph now reads (line 300): ‘In conclusion, both media tested under these conditions result in WD-PAEC cultures that possess several hallmarks of airway epithelium *in vivo* **and resulted in comparable experimental outcomes in several of the parameters assessed. However**, **our data also indicate that** the choice of medium used to differentiate and maintain primary airway epithelial cell cultures may impact the experimental outcomes and care should be taken in choosing medium for the intended work’

Reviewer 2 states: ‘*one cannot conclude it is proliferation rate because plating efficiency was not monitored. In other word some plates may had fewer cells initially attach to the matrix. But this was not measured*.’

**Response**: We apologise that this may have been unclear in the original submission, but the cell cultures from all donors were seeded at the same density in Promocell medium. Only at the time of air-liquid interface (ALI) initiation were half of the cultures (per patient) grown in Pneumacult medium. Therefore, the number of cells that adhere to the Transwells should be virtually identical as the seeding conditions are exactly the same. Additionally, the two different seeding densities do not affect the ultimate number of cells in the differentiated cultures. This indicates that Pneumacult continues to promote cell growth and proliferation post ALI, which we think is a very important point.

Reviewer 2 states: ‘*Thus the consistency of results among plates and among different days (replicates) was either not measured or not performed.’*

**Response:** Experimentation on each donor was performed independently and were not run in parallel. There is remarkable consistency in these experiments, even with the expected donor-to-donor variation as shown in figures 1B and 4 and in the additional data included above. Please also see the response the editor’s main point. We are confident, therefore, that the differences arising in our data, although minor, are due to the different media and are not random variations.

Reviewer 2 states: ‘*The above points should be addressed with more than one replicate and Z factor scores calculated to actually understand whether the differences seen were due to differences between media type or just random variation.*

Although not ideal for small data sets such as this, we have calculated the z factor scores (below). Although not strong due to small sample size, the z factor scores for total cell number and ELISA reinforce our statistically significant findings comparing Promocell and Pneumacult media. The negative z factor scores for other figures is indicative of non-significant and overlapping results between the different test groups.

| **Figure** | **figure description** | **Z-score** |
| --- | --- | --- |
|  |  |  |
| 1A | TEER | -9.5 |
| 1B | Cell number 5x10^4^ | 0.409 |
| 3C | Transwell staining uninfected 5x10^4^ (%) | cilia= -18.06; goblet= -5.138 |
| 4 | TCID_50_ 2x10^4^ | -0.79 |
| 4 | TCID_50_ 5x10^4^ | -4.568 |
| 5C | Transwell staining infected 5x10^4^ (%) | cilia= -8.809; goblet= -1.87; RSV= -3.452 |
| 6 | ELISA 5x10^4^ | 0.347 |
